# Eating Pattern and Nutritional Risks among People with Multiple Sclerosis Following a Modified Paleolithic Diet

**DOI:** 10.3390/nu12061844

**Published:** 2020-06-20

**Authors:** Tyler J. Titcomb, Babita Bisht, David D. Moore, Yashpal S. Chhonker, Daryl J. Murry, Linda G. Snetselaar, Terry L. Wahls

**Affiliations:** 1 Department of Internal Medicine, University of Iowa, Iowa City, IA 52242, USA; babita-bisht@uiowa.edu (B.B.); david-d-moore@uiowa.edu (D.D.M.III); terry-wahls@uiowa.edu (T.L.W.); 2 Department of Pharmacy Practice and Science, University of Nebraska Medical Center, Omaha, NE 68198, USA; y.chhonker@unmc.edu (Y.S.C.); dj.murry@unmc.edu (D.J.M.); 3 Fred and Pamela Buffett Cancer Center, University of Nebraska Medical Center, Omaha, NE 68198, USA; 4 Department of Epidemiology, University of Iowa College of Public Health, Iowa City, IA 52242, USA; linda-snetselaar@uiowa.edu; 5 Department of Extended Care and Rehabilitation Service Line, Iowa City VA Health Care System, Iowa City, IA 52242, USA

**Keywords:** multiple sclerosis, nutrient adequacy ratio, modified Paleolithic diet, fruits, vegetables, recommended dietary allowances

## Abstract

Preliminary studies suggest that a modified Paleolithic diet may benefit symptoms of fatigue in progressive multiple sclerosis (MS). However, this diet restricts the consumption of eggs, dairy, and gluten-containing grains, which may increase the risk of micronutrient deficiencies. Therefore, we evaluated the nutritional safety of this diet among people with progressive MS. Three nonconsecutive 24-h dietary recalls were collected from (*n* = 19) progressive MS participants in the final months of a diet intervention study and analyzed using Nutrition Data System for Research (NDSR) software. Food group intake was calculated, and intake of micronutrients was evaluated and compared to individual recommendations using Nutrient Adequacy Ratios (NARs). Blood was drawn at baseline and the end of the study to evaluate biomarker changes. Mean intake of fruits and vegetables exceeded nine servings/day and most participants excluded food groups. The intake of all micronutrients from food were above 100% NAR except for vitamin D (29.6 ± 34.6%), choline (73.2 ± 27.2%), and calcium (60.3 ± 22.8%), and one participant (1/19) exceeded the Tolerable Upper Limit (UL) for zinc, one (1/19) for vitamin A, and 37% (7/19) exceeded the chronic disease risk reduction (CDRR) for sodium. When intake from supplements was included in the analysis, several individuals exceeded ULs for magnesium (5/19), zinc (2/19), sodium (7/19), and vitamins A (2/19), D (9/19), C (1/19), B_6_ (3/19), and niacin (10/19). Serum values of vitamins D, B_12_, K_1_, K_2_, and folate significantly increased compared to respective baseline values, while homocysteine and magnesium values were significantly lower at 12 months. Calcium and vitamin A serum levels did not change. This modified Paleolithic diet is associated with minimal nutritional risks. However, excessive intake from supplements may be of concern.

## 1. Introduction

Multiple sclerosis (MS) is a chronic immune-mediated neurodegenerative disease characterized by the demyelination of neurons due to an inflammatory response caused by migration of T lymphocytes into the central nervous system [1]. The disease affects nearly one million people in the U.S. [2], and patients experience a variety of symptoms including pain, fatigue, and changes in vision, cognition, and movement [3]. Currently, there is no known single etiology of MS, but environmental factors are thought to be major contributors [4]. Disease-modifying drugs are associated with reduced frequency of relapse and delayed disease progression [1] but are costly [5] and have adverse side effects [6,7]. Thus, many people with MS (pwMS) seek alternative therapies, including specialized diets and supplements [8].

One such specialized diet being investigated for MS is a modified Paleolithic diet based on Wahls Paleo^™^ diet principles, which recommends a dietary pattern that includes a daily intake of three one-cup servings each of leafy green vegetables, sulfur containing vegetables, and intensely colored fruits and vegetables (FV) [9]. In addition, 6–12 ounces of meat and fish are recommended daily, as well as 16 ounces of fatty fish and 12 ounces of organ meat weekly. This specialized diet also excludes all dairy, gluten-containing grains, and eggs, and processed foods, added sugar, and trans-fat are to be avoided. Furthermore, fermented foods, seaweed and algae, and nutritional yeast are encouraged, and supplements including cod liver oil, methyl-folate, methyl-B_12_, and vitamin D_3_ are included based on individual needs.

In a single-arm open-label 12-month pilot study, a multimodal intervention included this modified Paleolithic diet in addition to targeted supplementation, strengthening and stretching exercises, stress reduction techniques, and neuromuscular electrical stimulation was associated with improved fatigue, quality of life (QoL), mood, cognitive function and lipid profiles in a progressive MS cohort [10,11,12,13]. Due to the multimodal nature of the trial, it is not possible to determine which outcomes were specifically related to the diet. However, two small follow-up randomized controlled trials found that the study diet alone was associated with improvements in fatigue, QoL, and motor function compared to usual diet control groups [14,15].

In the original pilot study, participants reported decreased intake of energy and carbohydrates while protein and total fat intake did not change from baseline [12]. The analysis of foods tracked with daily food logs indicated that participants increased FV intake from 1–2 at baseline up to 7–8 daily servings [13] and had excellent overall diet adherence [10]. The consumption of 7–8 daily servings of FV surpasses current recommendations [16]. However, the modified Paleolithic diet excludes several foods (dairy, gluten-containing grains, and eggs), which may increase the risk of micronutrient deficiencies. In a follow-up trial among relapsing-remitting MS (RRMS) patients, the modified Paleolithic diet was associated with higher serum total vitamin K compared to controls, while serum values of thiamin, folate, and vitamin B_12_ did not differ at three months [14], suggesting limited risk for these micronutrients. One survey found that pwMS who reported following a Paleolithic diet may be at risk for deficiencies of vitamins D and E [17]. In addition, experimental seven-day menu models of a similar Paleolithic-based elimination diet theoretically meet requirements for all micronutrients except vitamin D, calcium, potassium, and choline for most individuals and iron for women of childbearing age [18]. However, micronutrient intake among pwMS following the Wahls Paleo^™^ diet principles has not been reported. The aim of this work was to report food group and nutrient intakes from food and supplements of study participants consuming this modified Paleolithic diet and to identify nutritional risks.

## 2. Materials and Methods

### 2.1. Participants and Study Design

As part of a 12-month single-arm, open-label, multimodal intervention that included the modified Paleolithic diet, targeted supplementation, exercise, stress reduction, and neuromuscular electrical stimulation, daily food logs and three nonconsecutive 24-h recalls were obtained from participants (*n* = 19) with progressive MS (17 secondary progressive and 2 primary progressive) with moderate to severe disability (Expanded Disability Status Score range 3.5 to 8) to monitor study diet adherence. Potential participants were excluded for major comorbidities, significant cognitive dysfunction, psychiatric disorders, insulin therapy, and coumadin therapy. Additional participant characteristics, enrollment details, and inclusion/exclusion criteria have been previously published [10,19]. At baseline, participant mean ± SD age was 51.6 ± 6.4 years with 14.7 ± 8.7 years since MS diagnosis and median EDSS score of 6.5 (range 3.5 to 8). All participants’ procedures were approved by the University of Iowa Institutional Review Board and informed consent was obtained from all subjects according to the Declaration of Helsinki. The clinical trials protocol registration number for the original trial is NCT01381354, and primary and secondary outcomes have been previously published [10,11,12,13].

### 2.2. Dietary Recalls and Analysis

Dietary recalls were conducted in the final months of the 12-month study by a trained Registered Dietitian and were assessed using Nutrition Data System for Research (NDSR) software (Nutrition Coordinating Center, Minneapolis, MN, USA). Food group intake was assessed to establish dietary patterns and assess group and individual adherence to the study diet. The weight of each food item obtained from NDSR was standardized to FoodData Central (formerly the United States Department of Agriculture National Nutrient Database) standard serving size weights for each respective food item to obtain estimated food group intake in cup, ounce, teaspoon, or tablespoon equivalents [20]. Dietary recalls were compared to food groups reported with daily food logs to ensure accuracy.

Dietary intake of micronutrients from food and supplements was assessed for inadequate or excessive intakes at the individual level. Mean micronutrient intakes from food were calculated from the three 24-h recalls for each individual and compared to the respective Recommended Daily Allowance (RDA), or Adequate Intake (AI) for micronutrients with no established RDA, for each micronutrient to calculate Nutrient Adequacy Ratios (NAR). Mean Adequacy Ratios (MAR) were then calculated for each individual by averaging NARs that were truncated at 100%. Individual MARs were combined to evaluate overall nutritional adequacy of the modified Paleolithic diet among pwMS. Intake of each micronutrient was compared to respective Tolerable Upper Limits (UL) or Chronic Disease Risk Reduction (CDRR) intake levels to assess risk of excessive intake both with and without supplements.

### 2.3. Laboratory Assessment and Analysis

Blood was collected via venipuncture at baseline and every three months for one year to monitor serum metabolite changes and the safety of the dietary intervention. Serum calcium, magnesium, folate, homocysteine, and vitamins B_12_ were analyzed at Iowa City VA Health Care System, Department of Pathology. Serum samples were sent to the University of Nebraska Medical Center and the University of Wisconsin-Madison for analysis of vitamin K [21] and vitamin A [22], respectively. Data are represented as mean ± standard deviation (SD) or frequencies unless otherwise noted. Changes in serum metabolite concentrations from baseline to 12 months were assessed with two-sided paired *t*-tests (alpha ≤ 0.05) using SAS software version 9.4 (SAS Institute, Cary, NC, USA).

## 3. Results

### 3.1. Dietary Assessment

Study participants reported an average intake of recommended FV of 10.3 ± 4.5 servings/day, which was comprised of 6.3 ± 3.1 servings of intensely colored FV, 2.2 ± 1.4 servings of leafy green V, and 1.9 ± 1.4 servings of sulfur-rich V (Table 1). The assessment of three-day average intake indicated that 84% of participants (16/19) consumed the recommended three daily one-cup servings of intensely colored FV, 32% (6/19) consumed the recommended three daily one-cup servings leafy green vegetables, and 16% (3/19) consumed the recommended three daily one-cup servings of sulfur-rich vegetables. For the excluded food groups, 68% (13/19) reported not consuming dairy, 95% (18/19) avoided eggs, and 79% (15/19) avoided gluten-containing grains. The combined meat and fish intake was 5.8 ± 4.3 ounces daily which is below the study diet recommendation for 6 to 12 ounces daily. Thus, only 32% (6/19) met the recommended intake. Furthermore, only 16% (3/19) consumed a daily amount of fatty fish (weekly recommendation of 16 ounces), and no participants (0/19) consumed organ meat (weekly recommendation of 12 ounces), though three individuals did report organ meet consumption. For the remaining encouraged foods, 58% (11/19) and 21% (4/19) met the recommendations for seaweed/algae and nutritional yeast, respectively.

The three-day mean intake of macronutrients from food and supplements was 167 ± 62.4 g/day available carbohydrates, 79.7 ± 36.4 g/day protein, 91.7 ± 41.2 g/day fat, and 37.7 ± 15.7 g/d fiber for an average energy intake of 1820 ± 506 kcal/day (Table 2). In addition, the intake of most micronutrients from food was adequate compared to individual requirements. The combined MAR for all individuals was 91.5 ± 6.2%, indicating excellent micronutrient intake from food compared to individual requirements. NARs were above 100% for all micronutrients investigated except calcium (60.3 ± 22.8%), choline (73.2 ± 27.2%), and vitamin D (29.6 ± 34.6%; Table 3). Additionally, mean iron intake was 15.3 ± 5.33 mg/d, which is below the requirement of 18 mg/d for women of childbearing age. Despite NARs above 100%, many individuals did not meet individual recommendations for vitamin E (9/19), pantothenic acid (8/19), iron (6/19), and zinc (6/19). Intake from food by individuals exceeded the ULs for zinc (1/19), vitamin A (1/19), and 37% (7/19) exceeded the CDRR for sodium, but no individual intake of other micronutrients from food exceeded respective ULs.

All participants (19/19) reported consuming supplements (Supplemental Table S1). The inclusion of intake from supplements reduced the number of individuals who did not achieve the individual recommended intake for vitamin D from 18/19 to 2/19 (Table 4). In addition, supplement intake lead to minor reductions in the number of individuals who did not achieve individual recommendations for calcium (17/19), vitamin E (5/19), pantothenic acid (1/19), iron (4/19), and zinc (4/19). However, choline did not change. Supplement intake lead to increased number of individuals who surpassed the UL for vitamin D (9/19), vitamin C (1/19), niacin (10/19), vitamin B_6_ (3/19), magnesium (5/19), and zinc (2/19).

### 3.2. Laboratory Assessment

The baseline and 12-month serum concentrations of calcium, magnesium, folate, vitamins B_12_, D, K_1_, K_2_, A, and homocysteine are reported in Table 5. Serum calcium, total vitamin A, and retinyl esters did not change compared to respective baseline values. Folate increased from 15.8 ± 3.85 ng/mL at baseline to 18.8 ± 2.67 ng/mL (*p* = 0.022) and vitamin B_12_ increased from 784 ± 605 pg/mL to 1220 ± 582 pg/mL (*p* = 0.035). In addition, homocysteine decreased from 11.7 ± 6.62 μmol/L at baseline to 8.54 ± 1.51 μmol/L at 12 months (*p* = 0.048). Serum vitamin D increased from 42.7 ± 17.5 ng/mL at baseline to 59.5 ± 21.4 ng/mL at 12 months (*p* = 0.009), vitamin K_1_ increased from 0.74 ± 0.45 ng/mL to 1.22 ± 0.68 ng/mL (*p* = 0.022), and vitamin K_2_ increased from 1.57 ± 0.97 ng/mL to 2.23 ± 1.45 ng/mL (*p* = 0.050). Serum magnesium decreased from 2.15 ± 0.23 mg/dL at baseline to 2.07 ± 0.16 mg/dL at 12 months (*p* = 0.011).

## 4. Discussion

This analysis of 24-h dietary recalls demonstrates that pwMS consuming a modified Paleolithic diet based on Wahls Paleo™ diet principles increased fruit and vegetable consumption to greater than nine daily servings, which confirms previous self-reported daily food log findings [13] and exceeds current guidelines [16]. Study participants were more compliant with the recommendation for intensely colored fruits and vegetables than for leafy green or sulfur-rich vegetables, likely due to preference for the fruit included in this group. Furthermore, most participants were successful in eliminating gluten-containing grains, eggs, and dairy from their diet, which again is in line with previous self-reported findings [13]. Self-reported fruit and vegetable consumption and avoidance of gluten-containing grains were independently associated with reduced fatigue in this cohort [12].

Mean caloric intake was 1820 ± 506 kilocalories/day, which comprised approximately 38% from carbohydrates, 18% from protein, and 44% from fat. These macronutrient ratios are similar to those previously published from 2007 Harvard Food Frequency Questionnaires (FFQs) obtained from this cohort, but the energy intake is approximately 500 kilocalories/day higher compared to the FFQs [12]. However, it is important to note that FFQs are sets of prespecified questions that need to be tailored to each study group for accuracy [23]. Due to the common foods eliminated in this modified Paleolithic diet, it is likely that the FFQ used previously [12] did not adequately capture intake from less common foods such as organ meats, non-potato root vegetables, milk alternatives, and mushrooms that likely replaced the energy from excluded foods in this diet. Future studies using diets that eliminate common foods should consider this limitation of prespecified FFQs in adequately capturing less common foods that replace eliminated foods when assessing dietary intake.

Micronutrient intake from food surpassed individual recommendations for most vitamins and minerals However, vitamin D, choline, and calcium all had NARs below 100%, indicating obvious nutrients of concern. Low vitamin D status is associated with increased disease activity [24], which makes the inadequate vitamin D intake from food observed in this study alarming. However, the Healthy U.S.-Style Eating Pattern is also deficient in vitamin D [18], and vitamin D supplementation is recommended for pwMS [25]. Vitamin D supplementation increased intake to achieve individual recommendations for most individuals, which likely contributed to the higher serum vitamin D concentrations at the end of the study. In addition, serum calcium remained stable throughout the study period despite calcium intake below individual requirements for 95% (18/19) of this cohort. Inadequate calcium intake may exacerbate risk of osteoporosis, especially in those who are immobile or taking drugs that inhibit calcium absorption [26]. Because osteoporosis is a common comorbidity among pwMS, ensuring adequate intake of calcium may be beneficial, especially for those unable to engage in weight-bearing exercise [27].

Despite average intake above individual requirements, serum magnesium was significantly lower at 12 months compared to baseline values. Large-dose vitamin D supplementation is thought to deplete magnesium [28]. However, 12-month serum magnesium values were within the normal range in this study, so the clinical significance of this observation is not clear. Due to the necessity of magnesium for vitamin D activation and subsequent calcium absorption [29], ensuring adequate intake of magnesium may be beneficial to pwMS following a Paleolithic diet.

Many individuals did not meet individual recommendations for vitamin E (9/19). The inadequate intake of vitamin E in nearly half of the participants in this study is in agreement with previous findings among pwMS following a Paleolithic diet [17]. During treatment, higher serum vitamin E levels are associated with reduced odds of new lesion development [30], an observation supported by findings from animal studies [31,32]. However, it is unknown what impact inadequate vitamin E intake may have in MS.

Due to the recommendations for leafy green vegetables and organ meats, it is not surprising that the majority of participants achieved recommendations for vitamins A and K. Vitamin A supplementation is associated with reduced fatigue and depression in pwMS during treatment [33]. Serum retinol values have been associated with reduced odds of new brain lesion formation [34], reduced markers of inflammation [35], and increased brain volume [36] in MS. However, serum values did not change from baseline to 12 months in this study, likely due to hepatic regulation. In addition, serum values of vitamin K_1_ and K_2_ were significantly higher at 12 months compared to respective baseline values, confirming previous 3-month observations of total vitamin K among RRMS patients [14]. One study observed that higher serum vitamin K_2_ values were associated with reduced risk of relapse in RRMS [37]. However, whether the association occurs in progressive MS is not known.

The elevated baseline homocysteine observed in this study is a common clinical observation in MS patients [38], and the highest levels are associated with increased disease severity [39]. Elevated plasma homocysteine is suggestive of impaired transmethylation due to folate or vitamin B_12_ deficiency [40], which may impact myelin structure and remyelination due to decreased methylation of myelin basic protein [41]. A recent meta-analysis found significant supporting evidence that elevated homocysteine contributes to MS disease severity [42]. Serum folate and vitamin B_12_ significantly increased at 12 months compared to respective baseline values, which is in contrast to the previous diet-only randomized controlled trial 3-month observations [14]. The intake of folate and vitamin B_12_ were above individual recommendations in this study. However, this multimodal intervention included a targeted supplementation based on individual lab values [10] that included methyl-B_12_ and methyl-folate, which are more likely responsible for the increase in respective serum values and subsequent decrease in homocysteine values at 12 months.

Choline is necessary for remyelination [43] and has shown promise in animal models of MS [44], making the severe inadequate intake among this cohort alarming. Due to the limited food sources of choline, inadequate intake is common, and only 6.6% of adults meet the AI for choline in the U.S. [45]. Because 24-h dietary recalls do not adequately capture infrequently consumed foods such as liver, a rich source of choline recommended in this modified Paleolithic diet, it is possible that the mean intake of choline in this study is lower than actual intake. Eggs are a major dietary source of choline, providing approximately 150 mg per large egg. Since eggs were excluded in this modified Paleolithic diet intervention, pwMS who follow a Paleolithic diet may consider reintroducing egg, especially the yolks, into their diet if well tolerated to achieve adequate intake of choline.

Many individuals did not achieve recommendations for pantothenic acid (8/19) and iron (6/19). Pantothenic acid deficiency is rare and pwMS do not appear to have altered levels compared to healthy controls [46]. However, it is proposed to benefit pwMS through regulation of iron and oxygen transport [47]. Most of the participants who did not meet individual iron recommendations were women of childbearing age who have much higher recommendations. Thus, ensuring adequate iron intake may benefit women of childbearing age with MS [48]. In addition, intake of zinc was below recommendations among 6/19 participants. A meta-analysis of 13 studies found that MS patients have lower serum zinc compared to controls [49]. Animal studies suggest that low serum zinc may be due to MS pathogenesis-specific compartmental shifts in zinc [50]. Thus, it is unknown what affect inadequate intake may have on disease course.

The intake of most micronutrients from food did not surpass Uls. However, one participant exceeded the UL for zinc of 40 mg/day. The inspection of the food items reported in the 24-h recalls by this participant indicated that they had consumed 1.25 servings/day of a zinc-fortified smoothie that provided 34 mg (85% of the UL) of zinc each day. In addition, one participant exceeded the UL for vitamin A of 3000 μg/day due to 4 oz of beef liver reported on a single recall. The removal of these individuals from the dataset lowered NARs to 133 ± 57 and 234 ± 178 for zinc and vitamin A, respectively. However, the interpretation does not change, since NARs still exceed individual recommendations. In addition, seven participants exceeded the CDRR for sodium of 2300 mg, which is in contrast to experimental menu models of a similar diet [18]. Increased sodium intake may increase comorbidity risk [51] but does not appear to affect MS severity [52]. With the inclusion of supplements, additional concerns of exceeding ULs are raised for vitamins, D, C, B_6_, niacin, and the minerals magnesium and zinc. Long-term supplementation exceeding ULs may raise risk for negative health outcomes due to toxicity.

The modified Paleolithic diet used in this study was associated with adequate intake of most micronutrients and favorable metabolite changes among people with progressive MS. Targeted supplementation of specific micronutrients or inclusion of specific nutrient-rich foods may be beneficial for pwMS following a Paleolithic diet. However, efforts should be made to avoid long-term over supplementation. The small sample size of this study necessitates nutrient comparisons to individual recommendations. Therefore, this information needs to be interpreted with caution until a larger analysis allowing for population-level nutritional assessment with corresponding biochemical data can verify these findings. Another potential limitation is that the dietary recalls used in this analysis may be subject to recall bias due to the possibility of cognitive dysfunction among pwMS. However, the original trial excluded potential participants with severe cognitive decline [19] and observed that cognitive function improved over time [13], limiting the affects this potential limitation. This analysis of 24-h dietary recalls suggests that individual nutrient recommendations are achievable for most nutrients among pwMS adhering to this modified Paleolithic diet.

## Figures and Tables

**Table 1:** Daily intake of modified Paleolithic diet recommended, excluded, and encouraged food groups and the proportional of individuals meeting requirements (*n* = 19).

Food Group	Daily Intake (Mean ± SD)	Diet Recommendation	Individuals Meeting Recommendation (%)
**Recommended**			
Total FV (cup equivalents/day) ^1^	10.3 ± 4.5	6–9	16 (84)
-Intensely colored FV (cup equivalents/day) ^1^	6.3 ± 3.1	2–3	16 (84)
-Leafy green V (cup equivalents/day) ^1^	2.2 ± 1.4	2–3	6 (32)
-Sulfur-rich V (cup equivalents/day) ^1^	1.9 ± 1.4	2–3	3 (16)
**Excluded**			
Dairy (ounce equivalents/day)	0.2 ± 0.6	0	13 (69)
Eggs (egg/day)	0.0 ± 0.1	0	18 (95)
Gluten-containing grains (ounce equivalents/day)	0.0 ± 0.2	0	15 (79)
**Encouraged**			
Meat and fish (ounces/day)	5.8 ± 4.3	6–12	6 (32)
Organ meat (ounces/day) ^2^	0.2 ± 0.8	1.7	0 (0.0)
Fatty fish (ounces/day) ^2^	1.0 ± 1.6	2.3	3 (16)
Seaweed/algae (tsp/day)	2.2 ± 2.6	0.25	11 (58)
Nutritional yeast (tbsp/day)	0.6 ± 0.9	1	4 (21)

^1^ Recommendations for FV are higher for males. ^2^ Recommendations for organ meat and fatty fish are in amounts per week, thus the recommended daily amount displayed in the table represents the average daily serving required to achieve the weekly recommendation. Abbreviations: F, fruit; V, vegetable; tbsp, tablespoon; tsp, teaspoon.

**Table 2:** Energy and macronutrient intake from food and supplements determined from three 24-h dietary recalls obtained from individuals with progressive multiple sclerosis (MS) (N = 19) following the modified Paleolithic diet.

Nutrient	Intake (Mean ± SD)	% Kilocalories
Energy (kilocalories/day)	1820 ± 506	100.0
Available carbohydrate (g/day) ^1^	167 ± 62.4	38.2 ± 12.1
Added sugar (g/day)	19.9 ± 19.8	5.1 ± 6.2
Fiber (g/day)	37.7 ± 15.7	NA
Protein (g/day)	79.7 ± 36.4	17.9 ± 7.3
Total fat (g/day)	91.7 ± 41.2	43.9 ± 13.2
Saturated fat (g/day)	20.5 ± 14.1	9.8 ± 5.5
Monounsaturated fat (g/day)	34.5 ± 18.9	16.7 ± 7.7
Polyunsaturated fat (g/day)	29.2 ± 16.7	13.8 ± 6.1

^1^ Fiber subtracted from total carbohydrate. Note: NA indicates not assumed to majorly contribute to energy.

**Table 3:** Micronutrient intake and nutrient adequacy ratios determined from three nonconsecutive 24-h recalls of foods consumed by individuals with progressive MS following the modified Paleolithic diet recommendations (*n* = 19) ^1^.

Micronutrient	Intake (Mean ± SD)	RDA or AI *	Number below Requirement (%)	UL or CDRR *	Number above UL (%)	Nutrient Adequacy Ratio
Vitamin A (μg) ^2^	1910 ± 1880	700–900	3 (16)	3000 ^3^	1 (5)	258 ± 198
Vitamin D (μg)	4.44 ± 5.19	15	18 (95)	100	0 (0)	29.6 ± 34.6
Vitamin E (mg) ^2^	18.1 ± 10.6	15	9 (47)	1000 ^4^	0 (0)	121 ± 70.6
Vitamin K (μg)	688 ± 555	90 *–120 *	1 (5)	ND	NA	703 ± 598
Vitamin C (mg)	311 ± 182	75–90	0 (0)	2000	0 (0)	391 ± 229
Thiamin (mg)	4.10 ± 4.91	1.1–1.2	3 (16)	ND	NA	355 ± 411
Riboflavin (mg)	4.62 ± 5.05	1.1–1.3	1 (5)	ND	NA	386 ± 390
Niacin (mg) ^2^	55.4 ± 38.2	14–16	0 (0)	35 ^4^	0 (0)	373 ± 234
Pantothenic acid (mg)	6.09 ± 2.69	5 *	8 (42)	ND	NA	122 ± 53.8
Vitamin B_6_ (mg)	5.29 ± 5.20	1.3–1.7	2 (11)	100 ^4^	0 (0)	360 ± 321
Folate (μg) ^2^	616 ± 304	400	5 (26)	1000 ^4^	0 (0)	154 ± 76.0
Vitamin B_12_ (μg)	7.62 ± 9.24	2.4	4 (21)	ND	NA	318 ± 385
Choline (mg)	337 ± 135	425 *–550 *	16 (84)	3500	0 (0)	73.2 ± 27.2
Calcium (mg)	654 ± 246	1000-1200	18 (95)	2000–2500	0 (0)	60.3 ± 22.8
Phosphorus (mg)	1190 ± 342	700	2 (11)	4000	0 (0)	170 ± 48.9
Magnesium (mg)	451 ± 124	320–420	5 (26)	350 ^4^	0 (0)	133 ± 42.5
Iron (mg)	15.3 ± 5.33	8–18	6 (32)	45	0 (0)	165 ± 83.7
Zinc (mg)	13.5 ± 9.30	8–11	6 (32)	40	1 (5)	154 ± 107
Copper (mg)	2.74 ± 1.36	0.9	0 (0)	10	0 (0)	304 ± 151
Selenium (μg)	94.6 ± 52.9	55	2 (11)	400	0 (0)	172 ± 96.2
Potassium (mg)	4170 ± 1300	2600–3400 *	3 (16)	ND	NA	148 ± 43.5
Sodium (mg)	2030 ± 976	1500	5 (26)	2300 *	7 (37)	149 ± 67.4
Manganese (mg)	5.28 ± 1.57	1.8–2.3	0 (0)	11	0 (0)	277 ± 91.7
Mean Adequacy Ratio ^5^						91.5 ± 6.2

^1^ Dietary Reference Intake (DRI) range represent the range in individual nutrient requirements for the different life-stage groups of the participants included in this study. ^2^ As retinol activity equivalents (RAEs), alpha-tocopherol equivalents, niacin equivalents, and dietary folate equivalents for vitamin A, vitamin E, niacin, and folate, respectively. ^3^ Tolerable Upper Limit (UL) for vitamin A is only for preformed retinol and does not include RAEs provided by provitamin A carotenoids, thus intake of only preformed retinol was compared to the UL. ^4^ UL for vitamin E, niacin, vitamin B6, folate, and magnesium is only for synthetic or supplemental forms, thus intake of only these forms were compared to the UL. ^5^ Mean adequacy ratios determined for each individual by truncating nutrient adequacy ratios at 100% and calculating individual means. Data represent average among individuals. Note: ND indicates not determined, NA indicates not applicable, * indicates value refers to the AI for the micronutrient in the corresponding row.

**Table 4:** Micronutrient intake and nutrient adequacy ratios determined from three nonconsecutive 24-h recalls of food and supplements consumed by individuals with progressive MS following the modified Paleolithic diet recommendations (*n* = 19) ^1^.

Micronutrient	Number of Supplement Users (%)	Supplement Intake (Mean ± SD)	Total Intake (Mean ± SD)	RDA or AI *	Number below Requirement (%)	UL or CDRR *	Number above UL (%)
Vitamin A (μg) ^2^	6 (32)	376 ± 820	2290 ± 1860	700–900	1 (5)	3000 ^3^	1 (5)
Vitamin D (μg)	17 (89)	168 ± 323	173 ± 324	15	2 (11)	100	9 (47)
Vitamin E (mg) ^2^	10 (53)	35.8 ± 82.5	55.7 ± 82.8	15	5 (26)	1000 ^4^	0 (0)
Vitamin K (μg)	7 (37)	8.33 ± 13.9	696 ± 571	90 *–120 *	0 (0)	ND	NA
Vitamin C (mg)	14 (74)	299 ± 540	609 ± 562	75–90	0 (0)	2000	1 (5)
Thiamin (mg)	17 (89)	61.2 ± 59.9	65.3 ± 60.2	1.1–1.2	1 (5)	ND	NA
Riboflavin (mg)	17 (89)	68.7 ± 78.3	73.3 ± 78.2	1.1–1.3	0 (0)	ND	NA
Niacin (mg) ^2^	16 (84)	201 ± 273	257 ± 273	14–16	0 (0)	35 ^4^	10 (53)
Pantothenic acid (mg)	16 (84)	38.3 ± 52.5	44.4 ± 52.4	5 *	1 (5)	ND	NA
Vitamin B_6_ (mg)	16 (84)	35.7 ± 53.7	41.0 ± 54.3	1.3–1.7	2 (11)	100 ^4^	3 (16)
Folate (μg) ^2^	19 (100)	1120 ± 714	1730 ± 765	400	2 (11)	1000 ^4^	0 (0)
Vitamin B_12_ (μg)	19 (100)	1270 ± 1440	1280 ± 1440	2.4	0 (0)	ND	NA
Choline (mg)	5 (26)	14.1 ± 27.0	351 ± 161	425 *–550 *	15 (79)	3500	0 (0)
Calcium (mg)	12 (63)	206 ± 239	860 ± 246	1000–1200	17 (89)	2000–2500	0 (0)
Phosphorus (mg)	2 (11)	2.11 ± 6.19	1190 ± 385	700	2 (11)	4000	0 (0)
Magnesium (mg)	9 (47)	186 ± 354	637 ± 392	320–420	4 (21)	350 ^4^	5 (26)
Iron (mg)	5 (26)	0.79 ± 1.95	16.1 ± 5.75	8–18	4 (21)	45	0 (0)
Zinc (mg)	8 (42)	8.60 ± 12.8	22.1 ± 9.30	8–11	4 (21)	40	2 (11)
Copper (mg)	9 (47)	0.37 ± 0.74	3.11 ± 2.34	0.9	0 (0)	10	0 (0)
Selenium (μg)	4 (21)	11.3 ± 36.3	106 ± 77.9	55	2 (11)	400	0 (0)
Potassium (mg)	5 (26)	16.5 ± 30.9	4190 ± 1370	2600–3400 *	3 (16)	ND	NA
Sodium (mg)	7 (37)	12.2 ± 27.7	2040 ± 1150	1500	5 (26)	2300 *	7 (37)
Manganese (mg)	9 (47)	0.83 ± 1.07	6.11 ± 2.38	1.8–2.3	0 (0)	11	0 (0)

^1^ DRI range represent the range in individual nutrient requirements for the different life-stage groups of the participants included in this study. ^2^ As retinol activity equivalents (RAEs), alpha-tocopherol equivalents, niacin equivalents, and dietary folate equivalents for vitamin A, vitamin E, niacin, and folate, respectively. ^3^ UL for vitamin A is only for preformed retinol and does not include RAEs provided by provitamin A carotenoids, thus intake of only preformed retinol was compared to the UL. ^4^ UL for vitamin E, niacin, vitamin B6, folate, and magnesium is only for synthetic or supplemental forms, thus intake of only these forms were compared to the UL. Note: ND indicates not determined, NA indicates not applicable, * indicates value refers to the AI for the micronutrient in the corresponding row.

**Table 5:** Serum nutritional markers at baseline and 12 months among people with MS (*n* = 19) following the modified Paleolithic diet.

Metabolite	Reference Range ^1^	Baseline	12 Months	Change	*p*-Value ^2^
Calcium (mg/dL)	8.5–10.5	9.43 ± 0.36	9.52 ± 0.35	0.08 ± 0.39	0.35
Magnesium (mg/dL)	1.5–2.9	2.15 ± 0.23	2.07 ± 0.16	−0.08 ± 0.13	0.011
Folate (ng/mL)	3.0–20.0	15.8 ± 3.85	18.8 ± 2.67	3.86 ± 6.23	0.022
Vitamin B_12_ (pg/mL)	232–1245	784 ± 605	1220 ± 582	440 ± 841	0.035
Homocysteine (μmol/L)	<10.0	11.7 ± 6.62	8.54 ± 1.51	−3.65 ± 6.53	0.048
Vitamin D (ng/mL)	20–80	42.7 ± 17.5	59.5 ± 21.4	15.9 ± 24.1	0.009
Vitamin K_1_ (ng/mL) ^3^	0.10–2.20	0.74 ± 0.45	1.22 ± 0.68	0.35 ± 0.63	0.022
Vitamin K_2_ (ng/mL) ^3,4^	NA	1.57 ± 0.97	2.23 ± 1.45	0.48 ± 1.02	0.050
Vitamin A (μmol/L) ^5^	1.0–4.1	2.03 ± 0.52	1.95 ± 0.37	−0.08 ± 0.27	0.23
-Retinyl Esters (nmol/L) ^6^	<100.0	24.8 ± 25.9	21.8 ± 15.6	−3.10 ± 27.3	0.64

^1^ Presumptive upper and lower serum measure limits of healthy adults (normative laboratory values reported by Department of Pathology). ^2^ Determined by paired T-test comparing baseline to 12-month values. ^3^ Analyzed on a subset of *n* = 14 participants. ^4^ Sum of menaquinone-7 and menaquinone-4. ^5^ Sum of retinol and retinyl esters. ^6^ Sum of retinyl palmitate, oleate, laurate, and stearate. Reference range for retinyl palmitate only. Note: NA indicates not applicable.

## Supplementary Materials

The following are available online at https://www.mdpi.com/2072-6643/12/6/1844/s1, Table S1. Individual study participant supplement type, intake, and frequency.
