# Supplementary material for: Eating Pattern and Nutritional Risks among People with Multiple Sclerosis Following a Modified Paleolithic Diet
Source: Nutrients. 2020 Jun 20;12(6):1844. doi: 10.3390/nu12061844 (PMC7353368; doi:10.3390/nu12061844)
Supplement: Supplementary file 1 [file nutrients-12-01844-s001.pdf]

Table S1. Individual study participant supplement type, intake, and frequency

Participant	**Supplement Brand**	**Supplement Name (Dose)**	**Number of recalls reported**	**Doses per recall**
1	Jarrow Formulas	Co-Q10 (100 mg)	3	1
	Life Extension	L-Methylfolate (1000 μg)	3	1
	Source Naturals	Riboflavin (100 mg)	3	1
	Source Naturals	Thiamin (100 mg)	3	1
	Jarrow Formulas	Resveratrol (100 mg)	3	2
	Jarrow Formulas	Methyl-B_{12} (1000 μg)	3	1
	Jarrow Formulas	Niacinamide (500 mg)	3	1
	Pinnaclife	Mineral Boost	3	1
	Pinnaclife	Cleanse	3	1
	Pinnaclife	Essential	3	1
	Pinnaclife	Full Spectrum	3	1
	Pinnaclife	Micracle Olivamiine Omega-3 (2000 mg)	3	1
2	Centrum Silver	Ultra Women's	3	1
	Phillip's	Concentrated Milk of Magnesia	1	1
3	Dr. Christopher's	Memory Plus Formula	2	1
	Dr. Christopher's	Herbal Calcium Formula	2	2
	Dr. Christopher's	Nerve Formula	2	1
	Dr. Christopher's	Pancreas Formula	2	1
	Dr. Christopher's	Relax Eze	2	2
	Dr. Christopher's	Blood Stream Formula	3	1
	Dr. Christopher's	Lower Bowel Formula	2	1-3
	Dr. Christopher's	Bee Power Energy Formula	2	2
	Dr. Christopher's	Complete Tissue and Bone Tablet	2	2
	Jarrow Formulas	Methyl-B_{12} (1000 μg)	2	1
	Generic	Vitamin D_3 (50 μg)	2	2
	Generic	Echinacea & Goldenseal Root Plus	2	2
	Carlson	Norwegian Cod Liver Oil	1	3
	Now Foods	Plant enzymes	1	3
	Garden of Life	Immune Balance Yeast Freeze	1	2
	Garden of Life	Raw Probiotics Women 50 & Wiser	1	1
	Vitacost	Spirulina Powder	1	1
	Generic	Milk Thistle (250 mg)	1	1

	Standard Process Inc.	SP Cataplex B 1200	1	3
	Standard Process Inc.	Vasculin	1	3
5	Pinnaclife	Cleanse	2	1
	Pinnaclife	Micracle Olivamine Omega-3 (2000 mg)	3	3
	Mason	Vitamin B_{12} (1000 μg)	3	2
	Source Naturals	Thiamin (16 mg)	3	1
	Source Naturals	Riboflavin (18 mg)	3	1
	Generic	Alpha Lipoic Acid (300 mg)	2	1
	Jarrow Formulas	Resveratrol (100 mg)	2	1
	Generic	Coenzyme Q10 (100 mg)	2	1
	Nature's Way	Kelp Natural Iodine Source (660 mg)	3	2
	Generic	Klamath Blue Green Algae (500 mg)	3	4
	Walgreen's	Vitamin D (250 μg)	3	1
	Thorne	Methylfolate (1000 μg)	3	1
6	Swanson	Red Marine Algae (350 mg)	3	1
	Swanson	B-complex 50	3	1
	Generic	Vitamin D_3	3	4
	Procaps Laboratories	Coenzyme Q10 (100 mg)	3	2
	Source Naturals	Acetyl L-Carnitine (500 mg)	3	1
	Source Naturals	Riboflavin (100 mg)	3	1
	Jarrow Formulas	Creatine Monohydrate	3	1
	Jarrow Formulas	Resveratrol (100 mg)	3	1
	Pinnaclife	Micracle Olivamine Omega-3 (2000 mg)	3	2
	Pinnaclife	Essential	3	2
	Pinnaclife	Mineral Boost	3	1
	Jarrow Formulas	Taurine (500 mg)	3	1
	NOW	Thiamin (100 mg)	3	1
	Swanson	L-Methylfolate (1000 μg)	3	1
	Swanson	Methyl-B_{12} (1000 μg)	3	1
	Swanson	Niacinamide (500 mg)	3	1
	Pinnaclife	Full Spectrum	3	1

	Source Naturals	N-Acetylcysteine (600 mg)	3	1
9	Source Naturals	Riboflavin (100 mg)	3	2
	Source Naturals	Thiamin (100 mg)	3	1
	Jarrow Formulas	Co-Q10 (100 mg)	3	2
	Jarrow Formulas	Niacinamide (500 mg)	3	2
	Jarrow Formulas	N-Acetylcysteine	3	1
	Jarrow Formulas	Methyl-B_{12} (1000 μg)	3	1
	Jarrow Formulas	Taurine	3	1
	Jarrow Formulas	Alpha Lipoic Sustain (300 mg)	3	2
	Pinnaclife	Micracle Olivamine Omega-3 (2000 mg)	3	4
	Pinnaclife	Essential	3	1
	Pinnaclife	Full Spectrum	3	1
	Pinnaclife	Mineral Boost	3	1
11	Jarrow Formulas	Co-Q10 (100 mg)	3	1
	Life Extension	L-Methylfolate (1000 μg)	3	1
	Source Naturals	Acetyl L-Carnitine (500 mg)	3	4
	Jarrow Formulas	Alpha Lipoic Sustain (300 mg)	3	1
	Jarrow Formulas	N-Acetylcysteine	3	1
	Jarrow Formulas	Taurine	3	1
	Jarrow Formulas	Methyl-B_{12} (1000 μg)	2	1
	Pinnaclife	Micracle Olivamine Omega-3 (2000 mg)	3	2
	Pinnaclife	Essential	3	2
	Pinnaclife	Mineral Boost	3	1
14	Pinnaclife	Micracle Olivamine Omega-3 (2000 mg)	3	2
	Pinnaclife	Essential	3	4
	Pinnaclife	Full Spectrum	3	2
	Pinnaclife	Mineral Boost	3	2
	Jarrow Formulas	Methyl-B_{12} (1000 μg)	3	1
	Sam's Club	Vitamin D_3 (125 μg)	3	1
	Source Naturals	Riboflavin (100 mg)	3	2
	Source Naturals	Thiamin (100 mg)	3	1
	Jarrow Formulas	Co-Q10 (100 mg)	3	1
	Jarrow Formulas	Taurine (1000 mg)	3	1
	Jarrow Formulas	Niacinamide (500 mg)	3	1

	Jarrow Formulas	Alpha Lipoic Sustain (300 mg) with biotin (330 μg)	3	2
	Source Naturals	Acetyl L-Carnitine (500 mg)	3	2
	Source Naturals	N-Acetylcysteine (1000 mg)	3	1
	Jarrow Formulas	Creatine Monohydrate	3	1
	Pinnaclife	Cleanse	3	1
	Life Extension	L-Methylfolate (1000 μg)	3	1
15	Nature Made	Stress B-Complex with vitamin C and zinc	3	1
	Generic	Vitamin D_3 (50 μg)	3	1
	Vitamin Shoppe	Methyl-B_{12} (1000 μg)	3	1
	Spring Valley	Liquid Filled Absorbable Calcium	3	2
	Jarrow Formula	Methylfolate (400 μg)	3	1-2
	Osteo Bi-Flex	Glucosamine + Chondroitin + MSM	3	2
	Metamucil	Multihealth Fiber	3	6
16	New Pioneer Food Coop	Spirulina (500 mg)	3	10
	Vita Cost	Norwegian Salmon Oil (2200 mg)	3	2
	Nature Made	Flax Seed Oil	3	1
	Vita Cost	Ginkgo Biloba (60 mg)	3	2
	Vita Cost	Ginseng	3	2
	Nature’s Way	Kelp Natural Iodine Source (660 mg)	3	3
	Kyolic	Formula 102: Aged Garlic Extract	3	4
	Vita Cost	L-Taurine (850 mg)	3	1
	Generic	Creatine Monohydrate	3	1
	Hyvee	Vitamin C (1000 mg) with Rose Hips (37 mg)	3	1
	Vita Cost	Vitamin D_3 (125 μg)	3	1
	Nature Made	Vitamin E (450 mg)	3	1
	Vita Cost	Carotene complex (10000 IU provitamin A)	3	1
	Vita Cost	Methyl-B_{12} (5000 μg)	3	1
	Vita Cost	Methylfolate (800 μg)	3	1
	Vita Cost	B100 Complex	3	2
	Vita Cost	Coenzyme Q10 (400 mg)	3	1

	Standard Process Inc	Cardio Plus	3	2
	Best Process	Alkaline Super Digest	3	4
17	Generic	Vitamin D_2 (1250 μg)	1	1
	Superior Source	Methyl-B_{12} (1000 μg)	3	1
	Life Extension	L-Methylfolate (1000 μg)	3	2
	Kal	Magnesium	3	2
	Welby Health	Multivitamin	3	1
	NOW	Evening Primrose Oil	3	2
	Healthy Origins	Vitamin D_3 (60 μg)	3	3
	NOW	Ubiquinol (50 mg)	3	2
	NOW	Red Yeast Rice (600 mg)	3	6
	Source Naturals	Wellness Formula Herbal Defense Complex	3	10
	NOW	Quercetin with Bromelin	3	3
	MegaFoods	Balanced B Complex	3	1
	NOW	Cranberry concentrate	3	2
	Vitamin Shoppe	Calcium citrate + magnesium + vitamin D_2	3	4
	Heritage Natural Foods	Suprema Dophilus	3	1
	Arbonne Essentials	Digestion Plus	3	1
	Welby Health	Fish oil (1200 mg)	3	2
18	Jarrow Formulas	Methyl-B_{12} (1000 μg)	3	1
	Life Extension	Optimized folate	3	1
	Vital Choice	Vitamin D_3	3	1
19	Generic	Vitamin D (1250 μg)	3	1
	Nature Made	Super B-complex with vitamin C and folic acid	3	1
	Nature Made	Omega-3 fish oil (1200 mg)	3	1
	Generic	Methylfolate (1000 μg)	3	1
20	Generic	Vitamin D (125 μg)	3	1
	Jarrow Formulas	Methyl-B_{12} (1000 μg)	1	1
	Jarrow Formula	Methylfolate	1	1
	Hyvee	B Complex with vitamin C	1	1
21	Generic	Vitamin D_3 (125 μg)	3	1
	Twin Labs	Complex B-100	3	1
	Generic	Vitamin B_{12} (1000 μg)	3	1
	Jarrow Formula	Methylfolate (400 μg)	3	3
	Solgar	Dessicated liver	3	3

	Kirkland Signature	Fish oil (1000 mg)	3	1
	Generic	Calcium (600 mg) + vitamin D (10 μg)	3	1
	Metamucil	Multihealth sugar-free fiber	3	2
22	Country Life	Vitamin C complex (500 mg)	2	1
	Superior Source	Women's B-complex	2	2
	Superior Source	Vitamin B_{12} (5000 μg)	2	1
	Source Naturals	Sublingual vitamin B_6 (25 mg)	2	1
	Kal	Niacin (50 mg)	2	1
	Superior Source	Vitamin D_3 (125 μg)	2	2
	Jarrow Formula	Methylfolate (400 μg)	2	1
	Ethical Nutrients	Malic magnesium + thiamin + vitamin B_6 + manganese + malic acid	2	1
	Dr Ron's	Organ delight	2	3-4
	Green Pasture	Blue ice fermented cod liver oil	3	2
	Dr Ron's	Doc's friendly flora probiotic	2	1
	Country Life	Coenzyme Q10 (30 mg) with vitamin E	2	1
26	Progressive Laboratories	Vitamin B_{12}	3	1
	Metagenics	Glycogenics B complex formula	3	2
27	Nature Made	Balanced B-100 B-complex	3	1
	Vitality Herbs & Clay	Sacred clay	1	2
28	Jarrow Formulas	Methyl-B_{12} (1000 μg)	3	1
	Generic	Vitamin D (125 μg)	3	1
	Solaray	B-complex 100	3	1
	Thorne	Methylfolate (1000 μg)	3	1
	Solgar	Desiccated liver	3	3
	New Chapter	Zinc food complex	3	1
	Nature's Way	Olive leaf	3	2
	Nature's Way	Astragalus root (470 mg)	3	2
	Nature's Way	Primadophilus optima	3	1
